# Acute Genetic Damage Induced by Ethanol and Corticosterone Seems to Modulate Hippocampal Astrocyte Signaling

**DOI:** 10.1155/2024/5524487

**Published:** 2024-02-26

**Authors:** Ana Laura Reyes-Ábalos, Magdalena Álvarez-Zabaleta, Silvia Olivera-Bravo, María Vittoria Di Tomaso

**Affiliations:** ^1^Departamento de Genética, Instituto de Investigaciones Biológicas Clemente Estable-Ministerio de Educación y Cultura, Montevideo, Uruguay; ^2^Unidad de Microscopía Electrónica, Facultad de Ciencias, Universidad de la República, Montevideo, Uruguay; ^3^Departamento de Neurobiología y Neuropatología, Instituto de Investigaciones Biológicas Clemente Estable-Ministerio de Educación y Cultura, Montevideo, Uruguay

## Abstract

Astrocytes maintain CNS homeostasis but also critically contribute to neurological and psychiatric disorders. Such functional diversity implies an extensive signaling repertoire including extracellular vesicles (EVs) and nanotubes (NTs) that could be involved in protection or damage, as widely shown in various experimental paradigms. However, there is no information associating primary damage to the astrocyte genome, the DNA damage response (DDR), and the EV and NT repertoire. Furthermore, similar studies were not performed on hippocampal astrocytes despite their involvement in memory and learning processes, as well as in the development and maintenance of alcohol addiction. By exposing murine hippocampal astrocytes to 400 mM ethanol (EtOH) and/or 1 *μ*M corticosterone (CTS) for 1 h, we tested whether the induced DNA damage and DDR could elicit significant changes in NTs and surface-attached EVs. Genetic damage and initial DDR were assessed by immunolabeling against the phosphorylated histone variant H2AX (*γ*H2AX), DDR-dependent apoptosis by BAX immunoreactivity, and astrocyte activation by the glial acidic fibrillary protein (GFAP) and phalloidin staining. Surface-attached EVs and NTs were examined via scanning electron microscopy, and labeled proteins were analyzed via confocal microscopy. Relative to controls, astrocytes exposed to EtOH, CTS, or EtOH+CTS showed significant increases in nuclear *γ*lH2AX foci, nuclear and cytoplasmic BAX signals, and EV frequency at the expense of the NT amount, mainly upon EtOH, without detectable signs of morphological reactivity. Furthermore, the largest and most complex EVs originated only in DNA-damaged astrocytes. Obtained results revealed that astrocytes exposed to acute EtOH and/or CTS preserved their typical morphology but presented severe DNA damage, triggered canonical DDR pathways, and early changes in the cell signaling mediated by EVs and NTs. Further deepening of this initial morphological and quantitative analysis is necessary to identify the mechanistic links between genetic damage, DDR, cell-cell communication, and their possible impact on hippocampal neural cells.

## 1. Introduction

Astrocytes are nonneuronal cells of ectodermal origin that sustain CNS homeostasis at all levels and provide for its defense against injury but also have a critical contribution to neurological and psychiatric disorders. As pivotal responders to all forms of CNS insults, the response of astrocytes to each specific damage condition may involve the loss of protective functions or gaining of neurotoxic properties [1–7]. Thus, neuroprotective or deleterious actions of astrocytes in each specific context will depend not only on the time and type of injury but mainly on the changes elicited in gene expression, morphology, proliferation, functions, and/or signaling [2, 6]. Such diversity of astrocyte responses implies an extensive signaling repertoire that includes gap junctions, nanotubes (NTs) [8–10], soluble factors, and extracellular vesicles (EVs) [1, 11, 12]. All of these allow astrocytes to be proposed as secretory cells with significant action on themselves (autocrine communication) or on the other neural cells (paracrine communication) [11, 13, 14].

Cocucci and Meldoles [15] reported that astrocytes released EVs that include exosomes (50–100 nm diameter) and ectosomes or microvesicles (>1,000 nm diameter) generated from early, late, and multivesicular endosomes that fuse with the plasma membrane or by a direct outward budding plasma membrane, respectively, to shed into the extracellular space. In addition, upon repetitive ATP stimulation, cultured astrocytes could release larger vesicles (1-8 *μ*m diameter) [16]. EVs may contain membrane proteins, lipids, signaling molecules, mRNAs, microRNAs, long noncoding RNAs, mtDNAs, growth factors, and cytokines [9, 16, 17]. These molecules could be involved in neural protection [17, 18] or in promoting damage as occurs either in glioblastomas [19, 20] or in some neurodegenerative diseases [13, 17, 21, 22]. In turn, astrocytes are also targets of EVs from sources other than neural cells, as demonstrated by the inflammatory response resulting from exposing them to EVs from human T-cell lymphotropic virus type 1, the blood-borne pathogen that is the etiological agent of T-cell leukemia/lymphoma in adults [23]. Since EVs are involved in various physiological and pathophysiological brain processes, they have begun to be used as biomarkers of normal and pathological situations [24].

Astrocyte NTs range from 50 to 200 nm, but they can reach ~700 nm [8] and enable cell-to-cell communication up to ~500 *μ*m [9, 25]. Although its formation in significant amounts during healthy conditions is debatable, astrocyte NTs are induced by oxidative stress [26], serum depletion, p53 activation, acidic microenvironment, or hypoxia (reviewed by [9]). In addition, microtubes are thicker cell-connecting tubes (wider than 0.7 *μ*m), share many features with NTs, and were described in gliomas of astrocytic origin [9, 27]. Microtubes contain actin microfilaments and microtubules, which support intercellular cargo transport and contribute to their apparently longer lifespan compared to NTs [9]. It has been proposed that microtubes could connect pathological cells such as those from glioblastoma with normal astrocytes [9] to widespread disease-associated molecules larger than those transported by NTs. Both nano- and microtubes seem to play important roles in many physiological and pathological cellular processes, through the establishment of “open conduits” that seem able to transport ions, organelles, or molecules, helping to synchronize cells, induce cell differentiation, or spread CNS cancers or neurodegenerative diseases [8–10].

On the other hand, it is widely accepted that injuring conditions may alter astrocyte EVs and NTs. It has been reported that in cultured astrocytes, besides altering GFAP levels and the cytoskeleton [28], proliferation, trafficking, oxidative stress, and survival [19, 28–30], ethanol (EtOH) increased the EV secretion and consequently their content of inflammatory-related proteins [31]. The same authors demonstrated that the EVs primed from EtOH-treated astrocytes could alter the physiological state of neurons likely contributing to the spread of neuroinflammation and development of apoptosis. On the other hand, patients submitted to stress increased astrocyte EV levels [12]. In this regard, exposure of hippocampal astrocytes to high corticosterone (CTS) concentrations employed to mimic stress-like conditions (100 nM and 1 *μ*M for 3 h) increased EV release [32]. This EV astrocyte response will impact on brain homeostasis and in the overall stress response, in view of the critical role that the hippocampus plays in this process. The same study also reports that the modulatory effects of CTS on astrocytic vesicular release imply significant changes in actin cytoskeleton and microfilament rearrangements [32]. In summary, the existing literature clearly evidences that EVs and NTs participate in the responses of astrocytes to injuries. Moreover, both modalities are considered key factors in propagating astrocyte signals. However, there is little evidence on the possible association between astrocyte genetic damage and early changes in the EV and NT repertoire and whether one or both specializations could be modulated to limit CNS damage.

In addition despite that EtOH [33, 34] and CTS [35, 36] can be genotoxic, no previous reports investigated whether the DNA damage induced by very short exposures to EtOH and/or CTS could change the communication repertoire of astrocytes in terms of NT emergence and EV formation and release. Moreover, no studies with similar aims were made in main brain regions related to memory and learning, such as the hippocampus [37, 38], which is also very important in the development and maintenance of addiction to widely abused drugs including alcohol [39] and in mood associated disorders [40].

By using an experimental paradigm consisting of exposing hippocampal astrocytes to 400 mM EtOH and/or 1 *μ*M CTS for 1 h [41], we analyzed whether the induced DNA damage and the initial stage of DNA damage response (DDR) could elicit significant changes in the surface-attached EV and NT astrocytic repertoire in terms of morphology and quantity. We decided to study hippocampal astrocytes since it is known that alcohol affects hippocampal functions such as memory and learning through mechanisms that involve astrocytes [42] and because CTS can affect hippocampal astrocytes as was reported in models of major depressive disorders [43]. The short-term exposure was selected to know if very short exposure elicits not only DNA damage but also a fast cell response (DDR), which was reported to restore genome integrity and preserve its stability [44–47]. The short exposure also helped us understand whether changes in the signaling repertoire of astrocytes represent a rapid cellular response. EtOH and CTS working concentrations were selected because both were the minor ones that elicited reliable DNA damage and DDR [41].

Genetic damage and DDR were assessed by analyzing the rapid phosphorylation of the histone variant H2AX (termed *γ*H2AX foci) around sites of DNA damage. *γ*-H2AX recruits a series of proteins involved in the downstream DDR pathway [48–53], including connections with the DNA repair [44, 54, 55]. To detect evolution of DDR and early signs of apoptosis, the DDR-related apoptosis, which operates via the regulation of the proapoptotic *bax* gene [56–58], was assessed by recognizing BAX (proapoptotic effectors BCL-2-associated X protein or BCL-2-like protein 4) immunoreactivity. Astrocyte morphology was evaluated by DIC, immunostaining against the glial acidic fibrillary protein (GFAP), and phalloidin labeling, and NTs and EVs on astrocyte surfaces were analyzed via scanning electron microscopy.

Our results revealed that the immunoreactivity against *γ*H2AX and BAX indicates that DNA damage and possibly the DDR cascade were induced. Besides, no morphological modification like astrocytic reactivity was detected. Interestingly, significant modifications of the EV and NT repertoires and different sizes, morphology, and complexity of the EVs were observed depending on the experimental condition.

## 2. Materials and Methods

### 2.1. Ethical Statement

This study was performed following the Principles of the Laboratory Animal Care, National Institute of Health of the United States of America, NIH, publication No. 85-23 (2011 revision) and with the No. 18611 Uruguayan Law that dictates the procedures for the use of animals in experimentation, teaching, and scientific research activities. Entire procedures were approved by the Ethical Committees for the Care and Use of Laboratory Animals (CEUA) of the Facultad de Ciencias-Universidad de la República and the Instituto de Investigaciones Biológicas Clemente Estable- (IIBCE) Ministerio de Educación y Cultura (CEUA-IIBCE No. 05/08/2016). All animal studies and protocols complied with the ARRIVE guidelines.

### 2.2. Animals

Forty male Wistar rats (1 day old) from Facultad de Ciencias-Universidad de la República were employed. Pregnant rats were grown in individual cages with food and water *ad libitum* at 23 ± 1°C and a 12 h light/dark cycle (07 : 00–19 : 00 h).

### 2.3. Primary Cultures of Hippocampal Astrocytes

Twelve independent cultures were performed by using 3 rat pups per culture. Procedures were carried out according to Olivera-Bravo et al. [59] with minor modifications. The rats were quickly decapitated under a laminar flow hood, brains dissected and placed in sterile PBS buffer, and meninges removed. Clean brains were transferred to another plate with sterile PBS, and the hippocampus was dissected and cleaned under a stereomicroscope. Then, pieces of clean hippocampi were located in sterile 15 ml Falcon with 1 : 10 volume of 0.05% trypsin-EDTA buffer and then incubated in a water bath at 37°C. After 25 min, trypsin was blocked by adding 3 ml of complete culture media composed of DMEM (Gibco, 12800082), +10% fetal bovine serum (FBS; Gibco, 12657011), and penicillin/streptomycin (Gibco, 15140122), and pipetted 7 times without bubbling. The cell homogenate was passed through a sterile 80 *μ*m sieve and centrifuged at 400 g for 10 min. The supernatant was discarded, and the pellet was resuspended in 1 ml of complete culture media. Then, the cells were counted, diluted at 400,000 cells/ml, seeded in 35 mm Petri dishes or 24 multiwell plates, and incubated at 37°C and 5% CO_2_. The complete culture medium was changed every day until confluence. Then, monolayers were gently agitated at room temperature (RT) and darkness for 48 h. A week after, cells were trypsinized and reseeded on slides with a standard-size 8 × 6 mm diameter Teflon reaction well with black background (Tef-Tek Micro Slides premium, PorLab) and 12 × 4 mm diameter glass coverslips (Citoglas®) for analysis by fluorescence microscopy or on Aclar film (Electron Microscopy Sciences) for scanning electron microscopy (SEM) analysis. Twenty-four hours before each experiment, the percentage of FBS was decreased by 2% to favor the quiescence of the culture.

### 2.4. Treatments and Experimental Conditions

Quiescent astrocyte cultures were treated for 1 h with 400 mM EtOH, 1 *μ*M CTS, or EtOH+CTS (400 mM and 1 *μ*M, respectively). For the controls, astrocytes were incubated in culture media (CM) or exposed to the CTS vehicle, dimethyl sulfoxide (DMSO) at 0.03% to prevent genotoxicity [60]. Each experimental condition (CM, DMSO, EtOH, CTS, or EtOH+CTS) was fulfilled in triplicate. The cultures were kept at 37°C with 5% CO_2_ during the exposure time, then washed in 10 mM, pH 7.4 PBS (3 times), and fixed according to the procedure to be applied later.

### 2.5. Indirect Immunocytofluorescence, Phalloidin Labeling, and Confocal Image Acquisition

Astrocytes from the different experimental groups were washed with 10 mM, pH 7.4 PBS at 37°C and fixed with 4% paraformaldehyde (PFA) for 15 min. Next, the cultures were washed 3 times with PBS (3 min each), permeabilized with 0.5% Triton X-100 for 20 min, and then nonspecifically blocked with 2% bovine serum albumin (BSA) for 30 min. After 3 washes with PBS, they were incubated with the corresponding antibodies: anti-*γ*H2AX (1 : 300; ab26350, Abcam, Cambridge, UK), anti-BAX (1 : 200; ab7977), or anti-GFAP (1 : 500; ab4674), in a humid chamber at 37°C for 30 min. Then, following 3 washes with PBS, the astrocytes were incubated for 30 min at 37°C, in a humid dark chamber with the secondary antibodies goat anti-mouse IgG Alexa Fluor 594 conjugated H&L (1 : 500; ab150116) and goat anti-rabbit IgG Alexa Fluor 488 conjugated H&L (1 : 500; ab150077). Next, 1.5 *μ*g/mL of 4′, 6-diamidine-2-phenylindole dihydrochloride (DAPI; D9542, Sigma-Aldrich Merck KGaA, Darmstadt, Germany) was used as the nuclear counterstain (20 min at RT) in all cases. After 2 washes with PBS, cells were mounted in ProLong Gold antifade (P36930, Invitrogen, Thermo Fisher Scientific, Waltham, MA, USA), and coverslip edges were sealed with colorless nail enamel.

To evaluate astrocyte morphology in the different experimental conditions, in a set of experiments, 1 : 250 dilutions of Alexa Fluor™ 633 Phalloidin (A22284, Invitrogen) were added together with 1.5 *μ*g/mL of DAPI during 20 min at RT. After 2 washes with PBS, cells were mounted and sealed as indicated above.

All preparations were preserved at 4°C, protected from light, and then imaged. Images were acquired under a Zeiss LSM 800 confocal microscope using a plan apochromatic oil immersion lens (63x, 1.4 NA) with 2x magnification and in sequential scan mode employing 405, 488, and 546 nm LASER lines. Images (voxel size: Δ*x*/Δ*y*/Δ*z* = 0.379/0.379/1.00 *μ*m) were saved with a resolution of 2048 × 2048 pixels, in .czi format, and then in noncompressed .tif format. Acquisition parameters were maintained among all the experimental conditions.

### 2.6. Sample Preparation for Scanning Electron Microscopy (SEM) Analysis and Imaging

Astrocyte suspensions were seeded on Aclar film as in Jiménez-Riani et al. [22] and Reyes-Ábalos et al. [41]. After a brief wash with warm PBS, cells from each experimental condition were fixed with 2.5% glutaraldehyde (4°C, 18 h), washed 3 times with PBS, postfixed with osmium tetroxide, and dehydrated with increasing EtOH concentrations (50%, 70%, 80%, 90%, and 100%, 5-10 min each). Solvent elimination was done with a dryer at a critical CO_2_ point to preserve intact internal structure, and pure gold metallization was carried out through a sputtering technique (gold plasma). Finally, samples were mounted in individual bronze dowels and submitted to SEM analysis. The astrocyte surface from each experimental condition was analyzed at ultrastructural levels by employing a SEM JEOL-5900-LV microscope. Images were obtained using secondary electrons at 20 mA with 300x, 1,000x, 2,000x, 3,000x, 10,000x, and 30,000x magnifications and saved in noncompressed .tif format. Image resolution in the *x*, *y* plane was 0.3 nm/pixel. Image sizes were 640 × 480 pixels, 8 bits, and 300k scan 3.

### 2.7. Image Processing and Data Collection

Digital confocal or SEM images were analyzed using FIJI (NIH) software. Different analyses were performed as described below.

#### 2.7.1. *γ*H2AX Focus Quantification on Confocal Images

To analyze the nuclear *γ*H2AX mark on confocal images, a digital command code was designed to work in batch format. This tool allowed executing blocks of actions in an automatic and agile way, working by folder of images and optimizing the processing and analysis time. The analysis executing code includes the following steps: (i) opening of .tif image files; (ii) channel splitting (green for GFAP labeling, red for *γ*H2AX foci marking, and blue for DAPI); (iii) 8-bit conversion with a pixel depth of 0-255; (iv) segmentation and generation of binary masks for the red channel; (v) definition of regions of interest (ROIs); (vi) storage of their coordinates in zip files .roi, to quantify *γ*H2AX foci using the 3D object counter plugin; (vii) segmentation, generation of binary masks for the DAPI channel, and delimiting nuclear ROIs; and (viii) counting nuclei (*n* = 100) per treatment using a 3D object counter plugin.

#### 2.7.2. Frequency, Diameter, and Length of NTs

On SEM micrographs obtained at 3,000x magnification, binary (Huang) masks of astrocytes (*n* = 25 per experimental point) were generated, from which NTs were counted using the FIJI cell counter plugin. The measurement of the length and diameter of NTs was performed employing the free-hand line tool of the FIJI program as follows: (i) a vector drawn from the edge of the cell soma to the end of each NT was used to measure the length, and (ii) to measure the diameter, a second vector perpendicular to the first one was drawn in each NT. The data were recorded in digital spreadsheets associating each astrocyte with the number, diameter, and length of its NT.

#### 2.7.3. Frequency and the Major Axis of EVs on Somas and NTs

On SEM images of astrocytes (*n* = 25 per experimental condition), taken at 10,000x or 30,000x magnifications, the characteristics of the EV surfaces were analyzed, and their numbers were quantified using the FIJI cell counter plugins. On previously obtained binary (Huang) masks, the major axis of EVs located on the somas of NTs was measured by drawing a vector along it, using the FIJI free-head line tool.

#### 2.7.4. Skeletonization

Skeletonization of SEM images showing NTs was made using the corresponding FIJI plugin on SEM images, as follows: (i) convert the image to 8 bits; (ii) apply despeckle, close the function, and remove outliers; (iii) save the image as a separate file; (iv) skeletonize; and (v) compare with the original figure.

### 2.8. Statistical Analysis and Illustrations

Using the GraphPad Prism 8® software (GraphPad Prism, RRID: SCR_002798), the Shapiro-Wilk test (*α* ≤ 0.05) was applied to check normal distributions concerning the following variables: (i) *γ*H2AX foci number, (ii) astrocyte areas, (iii) NT and EV frequencies, (iv) frequency of EVs on somas or NTs, (v) major axis of EVs, and (vi) length and diameter of NTs, all considered per astrocyte. Since none of them fit normal distributions, they were described employing the medians and 95% confidence intervals, as summary measures. Accordingly, differences between the distinct experimental conditions (CM vs. DMSO, CM vs. EtOH, DMSO vs. CTS, EtOH vs. CTS, EtOH vs. EtOH+CTS, and CTS vs. EtOH+CTS) were analyzed using the Kruskal-Wallis test with Dunn's test for multiple comparisons with *α* ≤ 0.05. 350-500 astrocytes from each experiment were analyzed. Since each condition was implemented in triplicate, medians (each corresponding to one outcome) were compared with each other, and data were pooled when *p* values were ≤0.05. Graphs were performed employing the GraphPad Prism 8® software and the figures using the Adobe Photoshop CC version 2017.

## 3. Results

### 3.1. EtOH and/or CTS Exposures Induced DNA Damage and DDR

**Figure 1:**
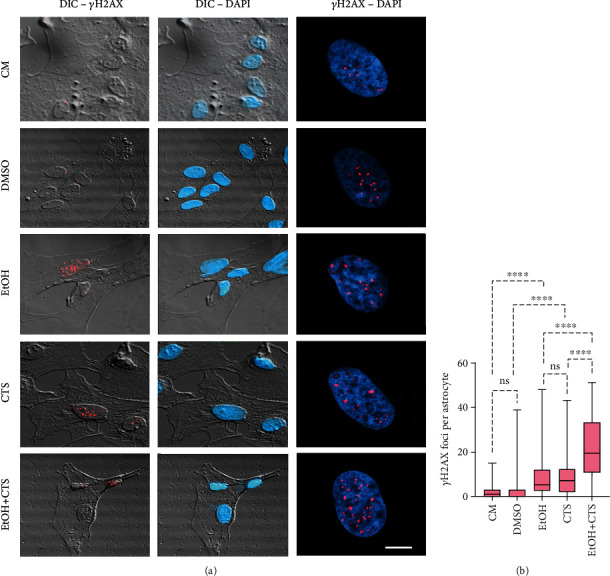
Primary DNA damage induced by EtOH and/or CTS in cultured astrocytes. (a) DIC and confocal images evidencing the DNA-damaged sites recognized as *γ*H2AX (red) foci in DAPI-stained nuclei (blue) as seen in confocal images on the right side of the panel. Left and central images depict *γ*H2AX and DAPI signals on DIC images, respectively. Calibration bar: 5 *μ*m. (b) Box plots show the number of *γ*H2AX foci per astrocyte in each experimental condition. Boxes enclose the data between the 25th and 75th percentiles. The median (50th percentile) is indicated by the cross line within each box. Differences between 100 astrocytes per analyzed variable and group were examined using the Kruskal-Wallis test with Dunn test for multiple comparisons. ^∗∗∗∗^*p* < 0.0001; ^ns^*p* > 0.05 (not significant). DIC: differential interference contrast; DAPI: 2-(4-amidinophenyl)-1H-indole-6-carboxamidine; CM: culture media; DMSO: dimethyl sulfoxide; CTS: corticosterone; EtOH: ethanol; EtOH+CTS: simultaneous EtOH and CTS treatment.

Analysis of the DNA damage induced by 1 h exposure to 400 mM EtOH and/or 1 *μ*M CTS assessed by DIC and confocal images of *γ*H2AX immunoreactivity (Figure 1) showed positive *γ*H2AX signals as bright (red) spots (termed foci) inside DAPI-stained nuclei (blue). *γ*H2AX foci were more abundant in the treated conditions relative to controls (Figure 1(a)), indicating rapid DNA damage and induction of DDR. As seen in DIC images, no *γ*H2AX signals were detected in the cytoplasm. In addition, quantitation of the number of *γ*H2AX foci per astrocyte (Figure 1(b)) confirmed significant frequency increases in EtOH or CTS compared to the respective controls with no differences between these conditions. The highest foci frequency corresponded to the combined EtOH+CTS group.

In addition, EtOH and/or CTS exposure did not elicit significant changes in astrocyte shape and reactivity when assessed by GFAP immunostaining and phalloidin-rhodamine labeling (Supplementary Figure 1). In all experimental conditions, GFAP immunostaining reflected fibrillary signals (green) that cover all astrocyte bodies and surround DAPI-positive nuclei (Supplementary Figure 1A). Regarding phalloidin labeling, it evidenced the strong F-actin astrocyte cytoskeleton and the geometric shape without the emission of significant cell processes (Supplementary Figure 1B).

### 3.2. Astrocytes Showed Increased Nuclear and Cytoplasmic BAX Immunoreactivity under Injured Conditions

**Figure 2:**
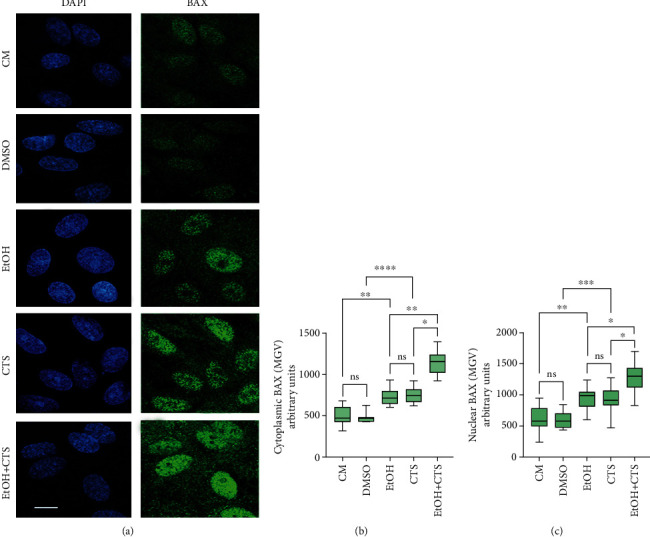
BAX immunoreactivity after astrocyte EtOH and/or CTS injuries. (a) Confocal images of control and injured astrocytes showing BAX immunostaining. Nuclei were labeled with DAPI (blue). Calibration bar: 10 *μ*m. (b, c) Distribution of BAX signals in the cytoplasm (b) and nucleus (c) of astrocytes from the different experimental conditions. The cross lines within the boxes represent the median. Differences between 100 astrocytes per group were assessed via the Kruskal-Wallis test with Dunn test for multiple comparisons. ^∗∗∗∗^*p* < 0.0001; ^∗∗∗^*p* < 0.001; ^∗∗^*p* < 0.01; ^∗^*p* < 0.05; ^ns^*p* > 0.05. DAPI: 2-(4-amidinophenyl)-1H-indole-6-carboxamidine; CM: culture media; DMSO: dimethyl sulfoxide; EtOH: ethanol; CTS: corticosterone; EtOH+CTS: EtOH and CTS cotreatment; MGV: mean grey value.

Representative confocal images obtained in all experimental conditions after recognizing BAX by specific immunolabeling showed predominant nuclear staining in control conditions and increased signals in both the cytoplasm and nuclei upon EtOH and/or CTS challenge (Figure 2). MGV analysis demonstrates that BAX signals increased in both the cytoplasm (Figure 2(b)) and the nuclear domains (Figure 2(c)) of astrocytes exposed to EtOH and/or CTS relative to their controls. In both compartments, the highest BAX MGV corresponded to the EtOH and CTS combined exposures, while no differences in individual EtOH or CTS treatments were found. Besides, it was observed that BAX signals tend to exclude DAPI-intense heterochromatic regions in all the experimental conditions.

### 3.3. EtOH and CTS Elicited Changes in the Intercellular Connections Associated with DNA Damage

**Figure 3:**
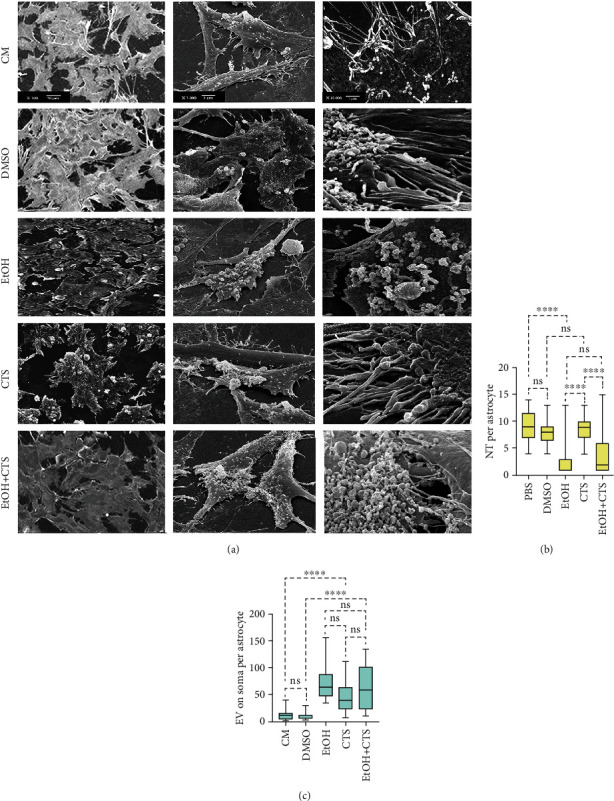
Astrocyte NTs and EVs related to EtOH and/or CTS exposures. (a) SEM images at three increasing magnifications showing NTs and EVs on the surface of astrocytes from control (CM and DMSO) or treated (EtOH, CTS, and EtOH+CTS) conditions. (b, c) Box plots depict the distribution of the number of NTs or EVs per astrocyte in each experimental condition. Differences between 50 astrocytes per variable and experimental group were analyzed employing the Kruskal-Wallis test with Dunn test for multiple comparisons. ^∗∗∗∗^*p* < 0.0001; ^ns^*p* > 0.05 (not significant). SEM: scanning electron microscopy; CM: culture media; DMSO: dimethyl sulfoxide; EtOH: ethanol; CTS: corticosterone; EtOH+CTS: EtOH and CTS coexposures; NTs: nanotubes; EVs: extracellular vesicles.

SEM images from all experimental conditions evidenced that astrocyte somas present many protrusions that extend from the cell margins to the substrate in arrangements where the length predominates over the cross-section and appeared very similar to the NTs previously described by Rustom et al. [61]. In addition, numerous variably shaped and sized formations derived from astrocyte membranes were identified as EVs appear loosely associated with cell margins and arranged on astrocyte surfaces in all experimental groups (Figure 3(a)). Remarkably, the frequency of NTs per astrocytes dramatically changed in the different experimental conditions, showing similar values in controls and CTS groups, but strongly decreasing in EtOH and less in EtOH+CTS (Figure 3(b)). Furthermore, the number of EVs was similar in astrocytes exposed to EtOH and/or CTS, but significantly higher than in controls (Figure 3(c)).

### 3.4. NT Main Morphological Parameters Seemed to Depend on the Injuring Challenge

**Figure 4:**
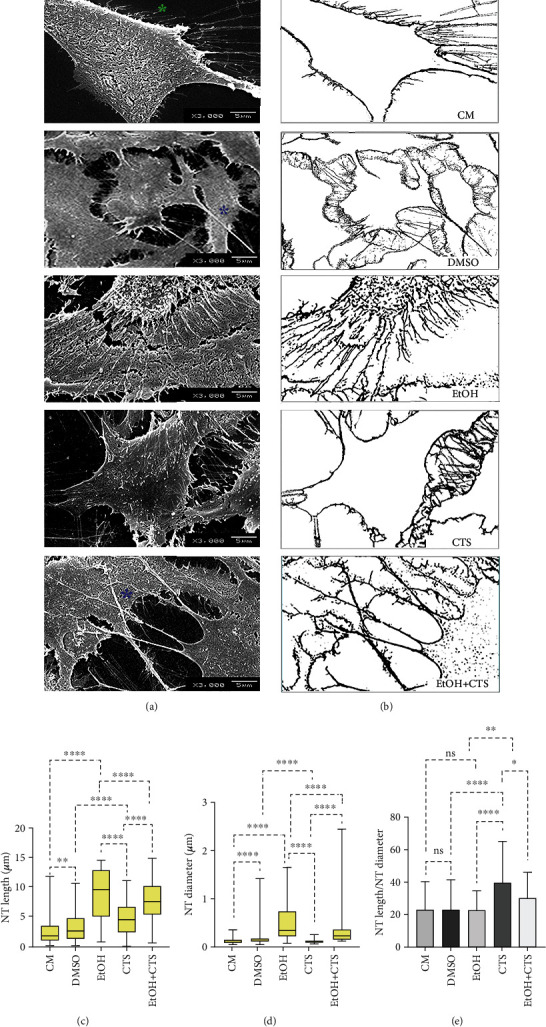
NT appearance, diameters, and lengths in controls and EtOH- and/or CTS-exposed astrocytes. (a, b) Panoramic SEM images and respective skeletonized masks illustrate examples of astrocyte NTs from controls (CM and DMSO), EtOH, and/or CTS. The green asterisk indicates NTs that softly decrease ending in a cell-free space, and blue ones are NTs that cross over the cell surfaces connecting different astrocytes. (c, d) Box plots represent the distribution of NT length and diameter in each experimental group. (e) Distribution of the ratio between NT length and diameter in each condition, using medians and 95% confidence intervals. Differences between 150 astrocytes per variable and group were examined via the Kruskal-Wallis with Dunn test for multiple comparisons. ^∗∗∗∗^*p* < 0.0001; ^∗∗^*p* < 0.01; ^∗^*p* < 0.05; ^ns^*p* > 0.05 (not significant). SEM: scanning electron microscopy; CM: culture media; DMSO: dimethyl sulfoxide; EtOH: ethanol; CTS: corticosterone; EtOH+CTS: simultaneous EtOH and CTS exposure; NTs: nanotubes.

Two main kinds of protrusions from the cell body could be distinguished (Figure 4(a)). One type is composed of short irregular protrusions of similar diameters at the point of emergence that softly decrease to end in a cell-free space (green asterisk in Figure 4(a)). The other formations are NTs of different lengths and diameters. In most cases, NTs seem to act as intercellular bridges between different astrocytes or cross over the cell surface to connect different cells (blue asterisks in Figure 4(a)). The respective skeletonized schemes evidence the two types of cell processes that generally appear in all experimental conditions (Figure 4(b)). Based on length measurement, the shortest NTs appear in both control conditions, whereas the longest were found in the groups where EtOH is present, with the EtOH+CTS group showing intermediate values between EtOH and CTS alone (Figure 4(c)). Regarding NT diameters, the biggest value was seen in the EtOH condition; the minimum, in the CTS group; and the intermediate, in the coexposed condition (Figure 4(d)). Therefore, the EtOH condition exhibits the longest and the thickest NTs. However, the assessment of the NT length/NT diameter ratios in absolute values indicates the biggest values in CTS followed by EtOH+CTS, with EtOH and controls appearing similar but lower than CTS (Figure 4(e)).

**Figure 5:**
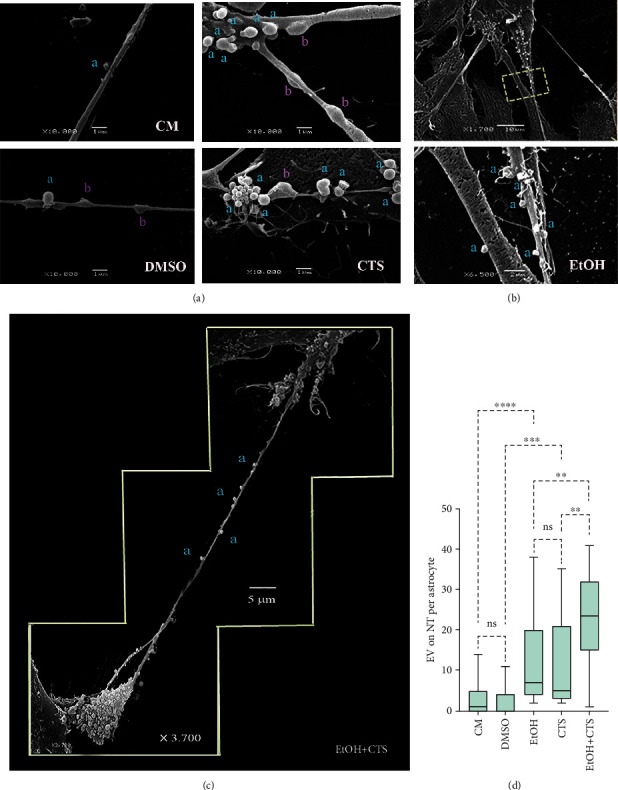
EVs on NT surfaces observed in control and in exposed astrocytes. (a) Representative SEM images showing NTs of different thicknesses with EVs located on the NT surfaces (indicated with “a” letters) or seemed to be inside the NTs (pointed to with “b” letters). (b) Two demonstrative SEM images were obtained at different magnifications, displaying NTs of different diameters with EVs attached to their surface. The dashed box depicted in the top image encloses the magnified area presented in the bottom image, to demonstrate the presence of EVs on the NT surface. (c) A composition of three SEM images showing a large NT connecting two hippocampal astrocytes. The EVs can be visualized on the NT at different distances from both cells. (d) Box plot representing the distribution of the number of EVs on NT per astrocyte of each experimental condition. Differences between 150 astrocytes per variable and group were analyzed via the Kruskal-Wallis test and Dunn test for multiple comparisons ^∗∗∗∗^*p* < 0.0001; ^∗∗∗^*p* < 0.001; ^∗∗^*p* < 0.01; ^ns^*p* > 0.05 (not significant). SEM: scanning electron microscopy; CM: culture media; DMSO: dimethyl sulfoxide; EtOH: ethanol; CTS: corticosterone; EtOH+CTS: EtOH and CTS cotreatment; NTs: nanotubes; EVs: extracellular vesicles.

In addition, SEM images revealed a significant number of EVs of rounded shapes associated with the cellular processes that seemed to be NTs (Figures 5(a)–5(c)). Many EVs appeared to be transferred between different cells via NTs (“a” letters in Figures 5(a)–5(c)). Some of them were disorderly arranged on the surface of the NTs, probably using it as a scaffold to move (“a” letters in Figures 5(a)–5(c)). Other EVs appeared to be inside the NTs (“b” letters in Figure 5(a)), which probably travel through the lumen of the NTs.

In spite that most of the NTs show the shapes and dimensions previously described, some NTs have doubled or tripled lengths when compared to the typical ones (Figures 5(a)–5(c)). Interestingly, the distribution of EVs in NTs similarly increased in the EtOH or CTS, being the highest augments in the coexposed astrocytes compared with the rest of the experimental groups (Figure 5(d)).

### 3.5. The Morphology and Size of EVs Depend on the Injuring Condition

**Figure 6:**
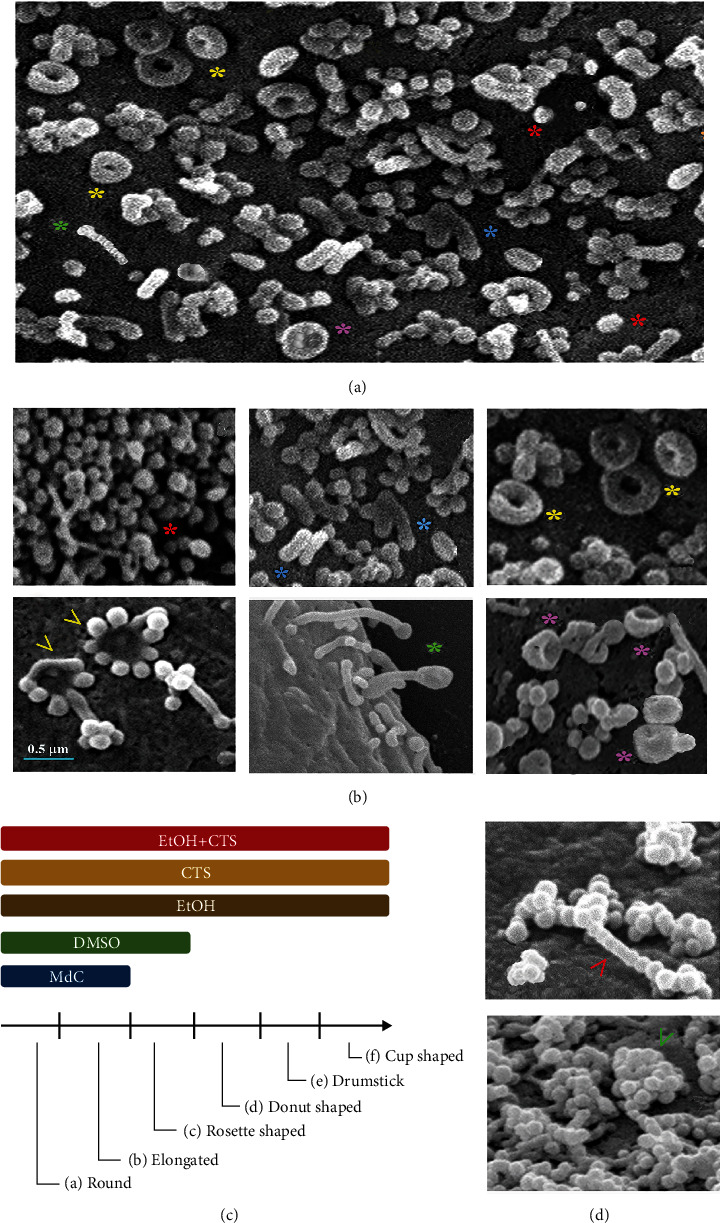
Distinctive appearance of astrocyte EVs related to control or exposure conditions. (a, b) SEM images depicting the shape and arrangement of EVs detected in controls or EtOH- and/or CTS-exposed astrocytes. Colored asterisks point to different EVs: red: round; blue: elongated; yellow: donut-shaped; green: drumstick-shaped; pink: cup-shaped; yellow arrowheads: rosette-shaped. (c) Diagram illustrating the EV forms observed on the astrocyte surfaces and their comparative distribution in the different experimental conditions (colored bars). (d) Ordered and disordered EV arrangements (red and green arrowheads, respectively). The phenotypic appearance of these EVs agrees, with minor differences, with the observation of Malenica et al. [23]. SEM: scanning electron microscopy; CM: culture media; DMSO: dimethyl sulfoxide; EtOH: ethanol; CTS: corticosterone; EtOH+CTS: EtOH and CTS coexposure; EVs: extracellular vesicles.

As observed in Figures 6(a) and 6(b), barely attached EVs of different shapes and dispositions were seen on the surface of cultured astrocytes. When using the EV classification reported by Malenica et al. [24] with minor modifications, we identified the following shapes: (i) round EVs that share a common spherical shape and similar size (red asterisks), (ii) less abundant elongated EVs of different lengths but with a clear main axis (blue asterisks), and (iii) donut-shaped EVs (yellow asterisks) in which both extremes appear very close or fused. Interestingly, there are donut-shaped EVs with central holes of different diameters, having the smallest and most compact appearance. The other types were (iv) rosette-shaped EVs (yellow arrowheads) that appeared as elongated vesicles adopting a “flower-like” arrangement with the base fused, (v) drumstick-shaped EVs (green asterisks) that seemed formed by an elongate vesicle with an enlarged round tip, and (vi) cup-shaped EVs (magenta asterisks) that appear as a rounded protrusion with a central depression giving it a cup-like appearance. Interestingly, a minor percentage of EVs showed intermediate morphologies that did not allow a clear classification inside one group. When analyzing the distribution of the morphology, controls showed EVs with predominantly round and elongated shapes, although in DMSO, some rosette-shaped EVs were found. Instead, in injuring conditions, all morphologies were observed (Figure 6(c)). EV arrangement was also variable, with most of the EVs appearing isolated, but others were orderly disposed in straight (red arrowhead) or random (green arrowhead) arrangements (Figure 6(d)).

**Figure 7:**
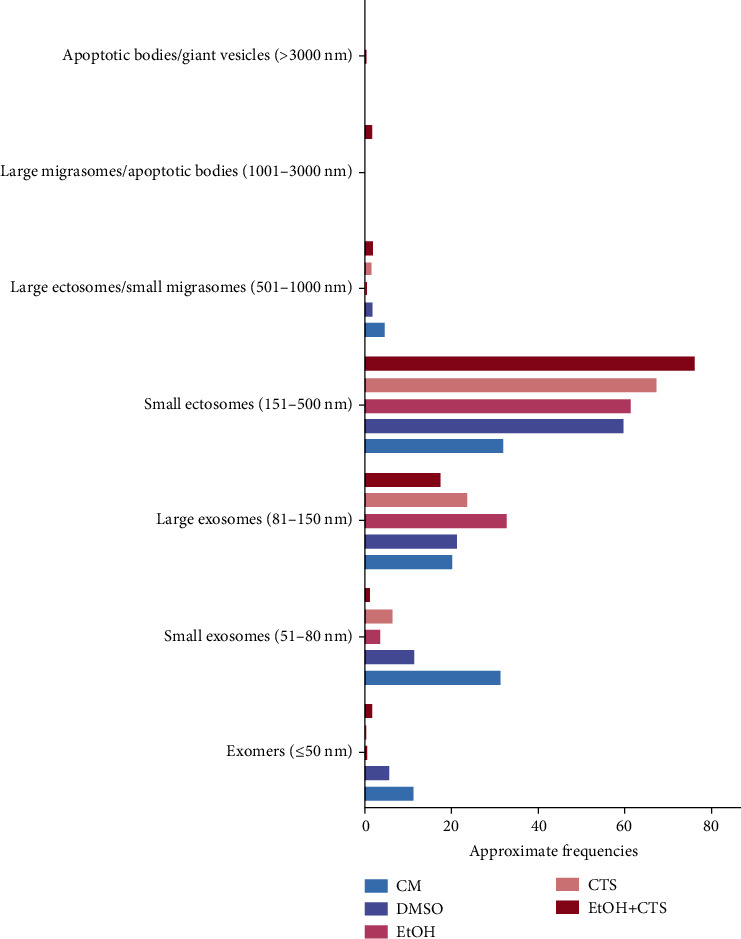
Frequencies of distinct EV morphologies in the different experimental conditions. The bar graphs represent the frequency of the different EV sizes and shapes observed on astrocyte surfaces according to Malenica et al. [24] and Di Daniele et al. [62], with minimum modification. The frequencies are expressed as approximate percentages of the total EVs per condition. Sizes are based on the length of their main axis. CM: culture media; DMSO: dimethyl sulfoxide; EtOH: ethanol; CTS: corticosterone; EtOH+CTS: EtOH and CTS cotreatment; EVs: extracellular vesicles.

Previous Malenica et al. [24] and Di Daniele et al. [62] reports were used to attribute a possible significance to EVs observed on astrocyte surfaces. Different EV subpopulations, have emerged when quantitation of EVs based on the major axis length and morphological features was performed (Figure 7). In all experimental conditions, the major axis of EVs compatible with small ectosomes was the most prevalent population, followed by those comparable to large exosomes. Frequencies of small ectosomes and large exosomes were higher in treated conditions than in controls, especially when compared to the CM condition. Controls showed higher frequencies of EVs compatible with small exosomes and exomers than treated conditions, with small exosomes prevailing in the CM control.

**Figure 8:**
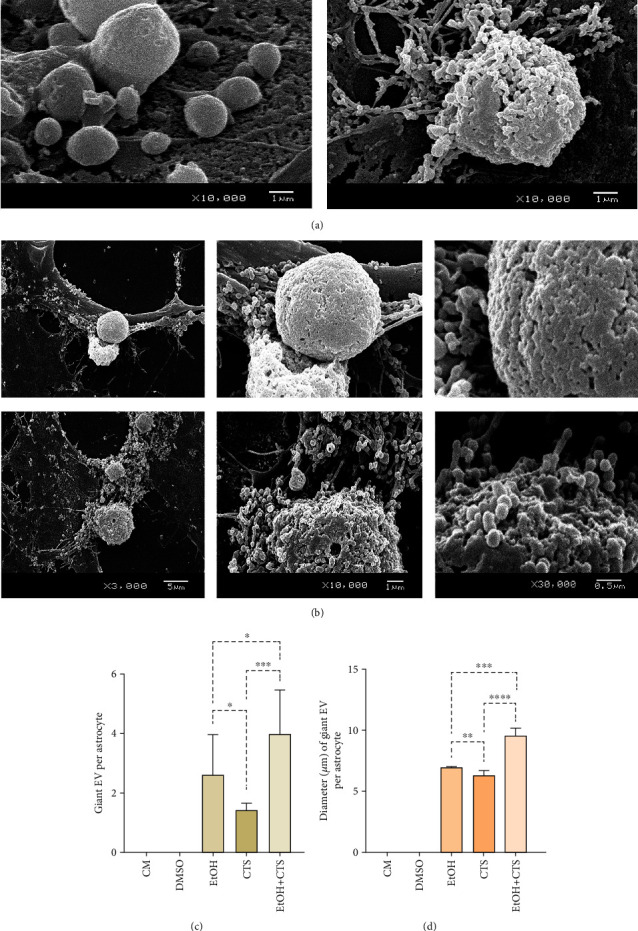
The largest EVs detected on astrocyte surfaces. (a) SEM images of EtOH and/or CTS conditions showing large EVs with distinct diameters and appearances that varied from smooth surfaces (left image) to heterogeneous and intricate morphology (right image). (b) SEM images obtained at three different magnifications exhibiting large EVs with surfaces showing different degrees of compaction and complexity. Meanwhile, in the bottom image, small EVs appear attached to the main formation. In the upper one, no individual EVs were observed on the surface. (c, d) Distribution of the number (c) and the main axis (d) (diameter in *μ*m) of the largest EVs (giant), per experimental condition, using medians and 95% confidence interval. Giant EV formations were observed only in EtOH- and/or CTS-exposed astrocytes. ^∗∗∗∗^*p* < 0.0001; ^∗∗∗^*p* < 0.001; ^∗∗^*p* < 0.01; ^∗^*p* < 0.05; ^ns^*p* > 0.05 (not significant). SEM: scanning electron microscopy; CM: culture media; DMSO: dimethyl sulfoxide; EtOH: ethanol; CTS: corticosterone; EtOH+CTS: simultaneous EtOH and CTS exposure; EVs: extracellular vesicles.

Regarding size, EVs ranged from ~20 nm up to ~8 *μ*m with more than 90% of them having a major axis lesser than 500 nm and ~95% being smaller than 1 *μ*m (compatible with exomeres, exosomes, and ectosomes). Less than 4% and 1% of total EVs were larger than 1 and 3 *μ*m, respectively (compatible with migrasomes and apoptotic bodies, respectively). Remarkably, >1 *μ*m and >5 *μ*m (termed as giant vesicles) EVs were exclusively seen in DNA-damaged astrocytes. Moreover, giant EVs showed particular features (Figure 8). Those complex formations exhibited considerable heterogeneity including differences in the external surface that could appear smooth or intricate with different degrees of compaction (Figures 8(a) and 8(b)). The number of the giant EVs (>5 *μ*m) and the lengths of their main axes were the highest in the EtOH+CTS condition (Figures 8(c) and 8(d), respectively).

## 4. Discussion

Our present data provided evidence of the sensitivity of the cell-signaling repertoire of murine hippocampal astrocytes to the genetic damage induced by acute exposure to the drug of human-wide consumption EtOH and/or the stress response hormone CTS (Figure 1). Genomic DNA damage was assessed by *γ*H2AX foci, which are produced a few minutes after its induction by the early phosphorylation of the H2A histone variant, H2AX [48–53]. *γ*H2AX immunoreactivity detected in astrocyte nuclei also indicated the early activation of the DDR cell pathways [41, 44–47]. The exclusive nuclear *γ*H2AX signal discarded significant damage in the mitochondrial DNA as evidenced by the absence of a cytoplasmic signal in all experimental conditions (Figure 1). *γ*H2AX foci signal double-strand breaks [52, 53] and also single-strand breaks caused by blockage of replication fork or by single-strand DNA intermediates of repair systems [63, 64]. Since double-strand breaks involve disruption of DNA continuity, they represent the most serious type of DNA lesions [65]. Therefore, our results evidence that significant astrocyte DNA damage was induced and that cell cycle control was immediately activated upon acute EtOH and/or CTS exposures. Moreover, under identical experimental conditions, we have recently detected changes in the immunoreactivity of the DDR effector cyclin-D1 and the excision repair endonuclease APE1 [41]. This finding suggests that, in the present experimental paradigm, the progression of the DDR cascade [66] and the activation of DNA repair [67] could also take place in DNA-damaged astrocytes.

However, the absence of modifications in the nuclear shape or chromatin compaction in treated astrocytes discards significant late apoptotic events, likely due to the short 1 h exposure. In this regard, DNA damage and DDR upon both injuring conditions occurred without significant morphological changes in astrocytes as assessed in DIC images (Figure 1) and for GFAP immunoreactivity (Supplementary Figure 1A). This result allows discarding significant astrocyte reactivity that is morphologically characterized by body shrinkage and protrusion of significant cellular processes [5]. In addition, as the actin cytoskeleton is the major determinant of cell morphology, the preservation of the astrocyte F-actin cytoskeleton as observed upon phalloidin labeling (Supplementary Figure 1B) indicates a preserved cytoskeleton in agreement with DIC and GFAP images. These results suggest that duration of the EtOH and/or CTS injury was not long enough to produce the previously reported actin disorganization [28, 68]. Therefore, the many functions dependent on the actin cytoskeleton would not be impaired despite significant DNA damage and DDR being induced.

Regarding Figure 2, it is interesting to note that BAX immunoreactivity increased in the cytoplasm and nucleus of astrocytes immediately after 1 h of EtOH and/or CTS exposures and that in both subcompartments, BAX signals paralleled the frequencies of *γ*H2AX foci. These findings suggest a relationship between the rapidly increasing BAX signal and injuring circumstances. However, as no morphological changes were observed in exposed astrocytes, changes in BAX immunoreactivity could precede detectable potential damaging effects.

Changes in BAX immunoreactivity could be linked to the activated DDR since BAX is an effector of the p53 DDR-dependent apoptosis [56–58]. The apoptotic functions of BAX are compatible with its increase in cytoplasmic and mitochondrial subcompartments [67–71]. However, BAX could shuttle from the cytosol to the nucleus during apoptosis in response to various stress stimuli. This occurs along with the translocation of some nuclear proteins such as p53 toward the cytoplasm where they could accomplish apoptotic roles or other functions [72, 73]. However, the consequences of BAX shuttling in the nucleus remained poorly elucidated and are still a debatable point [74, 75].

Our observation that BAX occupies euchromatic nuclear territories and tends to exclude silent heterochromatin regions suggests that it fulfills putative nuclear functions. In this aspect, Brayer et al. [76] showed that BAX was associated with chromatin in vitro in nonapoptotic cells, and that is linked with the modulation of the cell cycle and proliferation by modulating CDKN1A (cyclin-dependent kinase inhibitor 1A) that mediates the p53-dependent cell cycle G1-phase arrest in response to a variety of stress stimuli. Other functions attributed to nuclear BAX include modulation of basal differentiation and migration [76], which are critical aspects during tumorigenesis and CNS damage. Further studies to colocalize BAX with mitochondria will be necessary to understand whether BAX translocation to the mitochondria occurred in our experimental paradigm and to unravel if the detected early immunoreactivity of BAX precedes apoptotic cell death or is related to BAX non-proapoptotic functions.

Among other noncanonical BAX functions is the association with oxidative stress [73]. It has been described that EtOH intoxication elicits oxidative stress, proinflammatory mediators, and cytokine production that contribute to neuronal damage [77]. In addition, exposure to stress hormones such as CTS also facilitates the production of reactive oxygen species (ROS) [78–82] that can originate multiple DNA-oxidized products with altered bases and sugar moieties or broken strands [83]. In turn, cortical astrocytes treated with EtOH activate the inflammasome complex facilitating ROS generation [84]. Therefore, a significant imbalance between ROS production and removal, upon EtOH or CTS exposure, could challenge not only the astrocyte neuroprotective roles [85] but also their own cell proliferation and survival as previously shown in hippocampal astrocytes [34, 86–88]. Our results suggest that ROS-mediated DNA damage might contribute to astrocyte deleterious actions, either to themselves or to other neural cells.

However, the most remarkable findings of this work were the rapid changes on the astrocyte surface detected immediately after EtOH and/or CTS 1 h exposure, evidenced by clear modifications in NT and EV types and distribution, as well as in the diversity in quantity, morphology, and sizes observed in these membrane specializations. Obtained results strongly suggest that astrocyte signaling could be quickly modulated, as expected for these cells that are in charge of CNS homeostasis [5] and exhibit a plethora of cell communication strategies. It is known that under physiological or pathological circumstances, astrocytes communicate with other brain cells through NTs [9, 26, 61, 89, 90]. In our astrocyte cultures, we observed some NTs connecting with more or less distant cells, allowing direct communication between cells or facilitating the transfer of EVs on their surface and/or inside them, in some cases. The presence of exosomes and organelles, such as mitochondria within NTs, has been previously reported [91, 92]. NT development in cultured hippocampal astrocytes may depend on the activation of the p53 tumor suppressor gene [18], which is a central gene in DDR. As p53 regulates cell cycle control, DNA repair, senescence, or apoptosis pathways, when activated following DNA damage [93, 94], it indicates a connection between DDR and NT modifications. Remarkably, NT frequency decreases in astrocytes exposed to EtOH and/or CTS at the expense of increased EVs, suggesting a kind of interplay related to damage induction that will need to be further studied. Interestingly, we also found an inverse relation between NT and EV frequencies, presenting EtOH with the lowest NT and the highest EV frequencies and controls showing the opposite behavior. We could only speculate that different injury circumstances could differentially influence cell-signaling modalities, favoring long-distance signaling at the expense of cell-to-cell communication through NTs. Additionally, the production of EVs could be facilitated in some circumstances. In this sense, it has been reported that, as an initial step, EtOH increases the fluidity of biological membranes [95].

We realize that it is difficult to accurately classify a complex variety of EVs based solely on their size and feature, as aspects related to their composition, function, tissue of origin, and functional state would have to be considered ([96] MISEV2018 guide). Descriptions of several of these aspects can be seen in [24, 62, 97–109].

Nevertheless, we observed EVs related to the astrocyte surfaces of distinct sizes and features that agreed with previously published descriptions, especially the morphologies and classification reported by Malenica et al. [24] and Di Daniele et al. [62] (Figures 6–8). Even though the approach used (SEM) does not allow the confirmation of the EV type as transmission electron microscopy does, and need to be confirmed with complementary approaches, we can suggest based on its size and morphology that most of the EVs ranged from exomers (previously reported as nonmembranous nanoparticles) to small ectosomes in all conditions. However, the smallest EVs (exomers and small exosomes) predominated in controls since probably they are more associated with normal cell functions, while the largest ones compatible with large migrasomes (previously observed at the tips of retraction fibers of migrating cells), apoptotic bodies (formed during the late phase of apoptosis), and complex giant EVs seem to be associated with cellular response to strong injury since they were only identified, with a little frequency, in damaged conditions. We could also speculate that, given the role of astrocytes in maintaining CNS homeostasis, they would seem to activate mechanisms that would reverse DNA damage and its cellular consequences, allowing them to preserve their functions.

Different types of EVs are generated by distinct cellular processes, and EV composition reflects the physiological or pathophysiological state of the cells of its origin [24]. The complexity and heterogeneity of EVs observed on astrocyte surfaces could reflect the coexistence of different cellular events related to its production [97–99, 101, 103–107, 109]. They may be either the same mechanisms seen in controls, but at a higher degree of activation, or other mechanisms associated with damage circumstances that could operate [100, 102, 108] since the largest EVs were only observed in DNA-damaged astrocytes. In this regard, it is interesting to note that damaged nuclear and mitochondrial DNA were detected in EVs, suggesting that damaged DNA could be eliminated by integration into EVs [110].

Astrocytes play a prominent role in the protection of neurons against stressors [111–115]. In this respect, it has been reported that the astrocyte-conditioned medium protected hippocampal neurons against CTS-induced damage [85], suggesting that this depends on cell-cell communication. However, according to the stimuli received and the cellular microenvironment, astrocyte communication can have beneficial or detrimental consequences [4, 7, 43, 116]. There is evidence that cultured hippocampal astrocytes exposed to EtOH result in an intracellular redox imbalance and the expression of miRNA and proinflammatory molecules with augmented EV secretion [31, 117]. Increases in the production of EVs and calcium waves likely associated with damage were described in CTS-exposed astrocytes [32].

In response to different stimuli as those dependent on toxic protein aggregates or neuroinflammation, EVs can change their cargo causing oxidative stress, neuroinflammation, or synaptic dysfunction [17, 40, 43]. However, beneficial EV roles are also evident because they are involved in neuroprotection-modulating apoptosis, preserving neuronal function, and repairing neural damage [43]. Therefore, changes in signaling mechanisms of astrocytes can have a significant protective or harmful impact in neurons and other neural cells [4, 7, 116], and their potential impact on clinical aspects is increasingly considered [9, 27, 118].

Finally, the detected changes in the cellular communication pattern of astrocytes could be a not-yet-described part of the mechanisms involved in their response to genetic damage. Classically, DDR is considered a sequence of pathways that essentially operate at the nuclear and mitochondrial levels; however, the cells would respond as a single system. In this sense, it was reported that the local exposure of cells to low doses of ionizing radiation caused similar biological changes in cells not directly irradiated, giving the basis to the concept of a bystander response [119–122]. Later, the same effect was detected with other stress-inducing agents, proposing that the bystander effect depends on the release of chemical mediators, some transported as EV cargoes [123]. We hypothesize that the observed modulation of the NT and EV repertoires in injured astrocytes could indicate that the cellular response to DNA-damaging agents may trigger cellular mechanisms, in addition to the classical DDR pathways.

## 5. Conclusions

Our results demonstrated that hippocampal astrocytes responded to acute EtOH and/or CTS injuries, eliciting DNA damage, and possibly all canonical DDR pathways. Interestingly, an early modification in cell-cell communication processes was revealed, evidenced by changes in the pattern of EVs and NTs. We hypothesize that this rapid change in cell signaling could be a novel mechanism related to DDR. The reported quantitative and morphological analysis of EVs and NTs will be further confirmed and deepened to identify the connections of astrocyte genetic damage, with the evolution of DDR and cell-cell communicational patterns, and its impact on other neural cells and in the whole CNS.

## Data Availability

All data are included in the submitted manuscript.

## Supplementary Materials

Supplementary materials Supplementary Figure 1: GFAP immunostaining and phalloidin-rhodamine labeling of astrocytes. Confocal images showing typical astrocytes with nuclei labeled using DAPI, exhibiting fibrillary GFAP immunoreactivity (green) (A), or F-actin cytoskeleton phalloidin-rhodamine signal (red) (B) on astrocyte somas. Calibration bar: 10 *μ*m. DAPI: 2-(4-amidinophenyl)-1H-indole-6-carboxamidine; CM: culture media; DMSO: dimethyl sulfoxide; EtOH: ethanol; CTS: corticosterone; EtOH+CTS: EtOH and CTS coexposure.
