# Supplementary figures and images for: Acute Genetic Damage Induced by Ethanol and Corticosterone Seems to Modulate Hippocampal Astrocyte Signaling
Source: Int J Cell Biol. 2024 Feb 26;2024:5524487. doi: 10.1155/2024/5524487 (PMC10911912; doi:10.1155/2024/5524487)

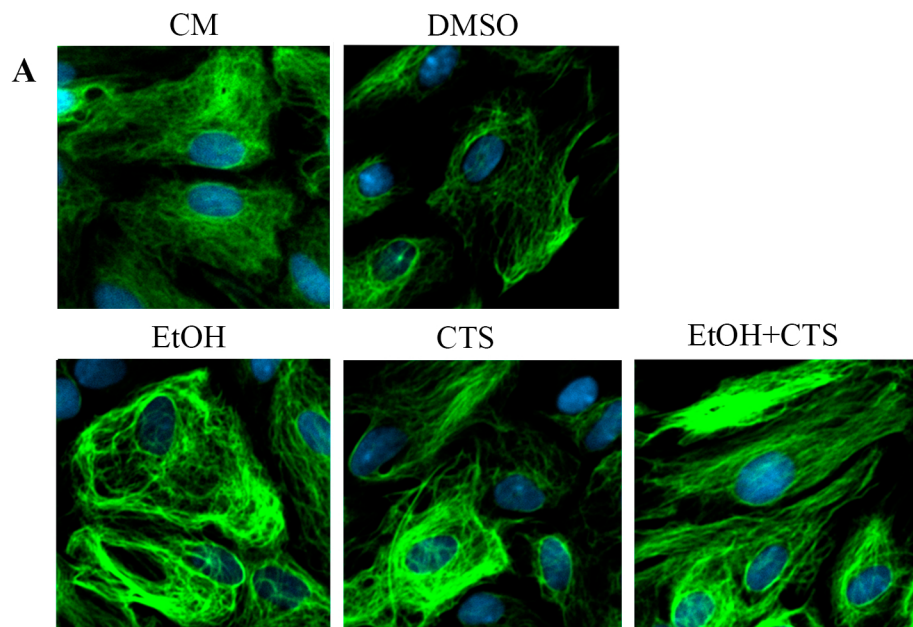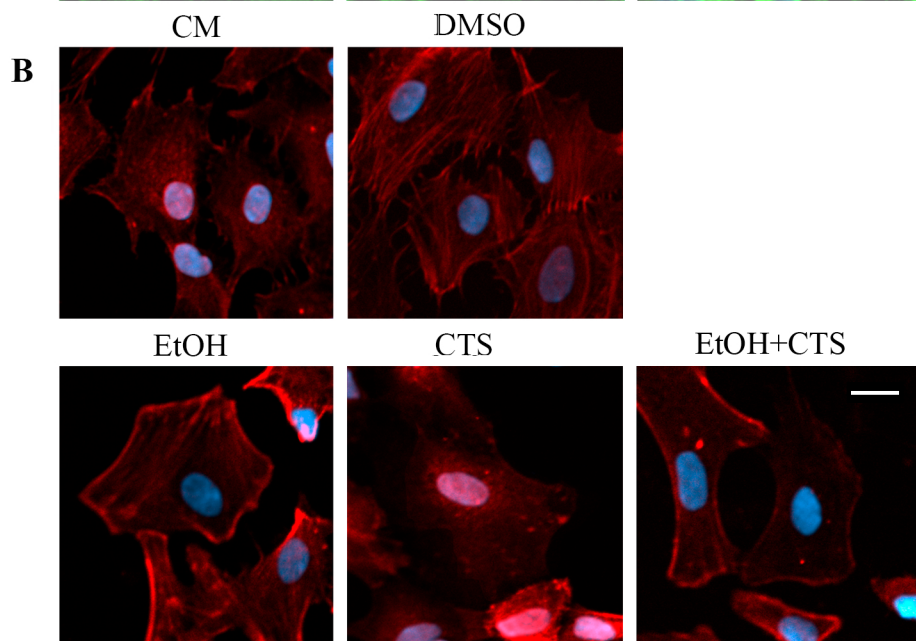

■ DAPI    
 ■ GFAP    
 ■ Phalloidin

Supplement: Supplementary materials — Supplementary Figure 1: GFAP immunostaining and phalloidin-rhodamine labeling of astrocytes. Confocal images showing typical astrocytes with nuclei labeled using DAPI, exhibiting fibrillary GFAP immunoreactivity (green) (A), or F-actin cytoskeleton phalloidin-rhodamine signal (red) (B) on astrocyte somas. Calibration bar: 10 μm. DAPI: 2-(4-amidinophenyl)-1H-indole-6-carboxamidine; CM: culture media; DMSO: dimethyl sulfoxide; EtOH: ethanol; CTS: corticosterone; EtOH+CTS: EtOH and CTS coexposure. [file 5524487.f1.pdf]
